# Circular High-Q Resonating Isotropic Strain Sensors with Large Shift of Resonance Frequency under Stress

**DOI:** 10.3390/s91209444

**Published:** 2009-11-25

**Authors:** Rohat Melik, Emre Unal, Nihan Kosku Perkgoz, Christian Puttlitz, Hilmi Volkan Demir

**Affiliations:** 1 Department of Electrical and Electronics Engineering, Bilkent University, Bilkent, 06800 Ankara, Turkey; E-Mail: rohat@ee.bilkent.edu.tr; 2 Nanotechnology Research Center, and Institute of Materials Science and Nanotechnology, Bilkent University, Bilkent, 06800 Ankara, Turkey; E-Mails: unale@bilkent.edu.tr (E.U.); kosku@bilkent.edu.tr (N.K.P.); 3 Department of Physics, Bilkent University, Bilkent, 06800 Ankara, Turkey; 4 Department of Mechanical Engineering and School of Biomedical Engineering, Orthopaedic Bioengineering Research Laboratory, Colorado State University, Fort Collins, CO 80523, USA; E-Mail: christian.puttlitz@colostate.edu

**Keywords:** RFIC, Q-factor, resonators, circular architecture, bioMEMS sensors, frequency shift, sensitivity

## Abstract

We present circular architecture bioimplant strain sensors that facilitate a strong resonance frequency shift with mechanical deformation. The clinical application area of these sensors is for *in vivo* assessment of bone fractures. Using a rectangular geometry, we obtain a resonance shift of 330 MHz for a single device and 170 MHz for its triplet configuration (with three side-by-side resonators on chip) under an applied load of 3,920 N. Using the same device parameters with a circular isotropic architecture, we achieve a resonance frequency shift of 500 MHz for the single device and 260 MHz for its triplet configuration, demonstrating substantially increased sensitivity.

## 1. Introduction

Fixation plates are routinely used for major bone fracture cases. As the healing tissue develops stiffness and strength, the load borne by the plate decreases [1]. During this process, a sensor capable of monitoring strain telemetrically and in real time is highly desirable. When force is applied to the sensor via its attachment to the fixation plate, the resulting strain is observed via a resonance frequency (f_o_) shift. Using this emerging technology, physicians would be able to assess the healing process by examining these temporal changes in strain.

Previously, we developed high quality factor (Q-factor) on-chip resonators [2] and demonstrated the proof-of-concept for utilizing the resonance frequency shift as an indirect measure of strain [3]. In this work, we significantly increased the sensor Q-factor and resonance frequency shift compared to the architectures used in the previous works. Here we present a circular architecture RF-MEMS bioimplantable strain sensor that demonstrates a substantially higher Q-factor and larger frequency shift compared to a rectangular architecture.

## 2. Design and Fabrication

**Figure 1.**
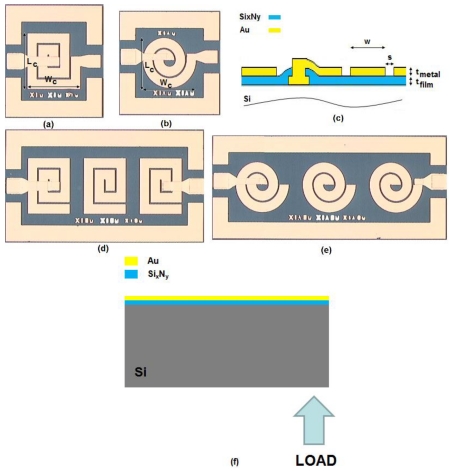
**(a)** Top-view single rectangular device, **(b)** top-view single circular device, **(c)** cross-sectional view of the device **(d)** top-view rectangular triplet configuration, **(e)** top-view circular triplet configuration, and **(f)** schematic illustration of the externally applied load.

For our resonators, we aim for a high Q-factor by using bio-compatible materials with a maximum possible resonance frequency shift. To design the resonator in a distributed spiral coil architecture with a high Q-factor (Figure 1), we consider the effects of substrate, dielectric material, dielectric thickness (t_film_), metal material, metal layer thickness (t_metal_), metal width (w) and spacing (s), number of turns (N), and area (W_c_ × L_c_) as explained in [2]. Further details of the formulas and techniques for Q-factor enhancement can be found in [4-13]. For biocompatibility, we choose to use silicon as the substrate, gold as the metal layer, and Si_x_N_y_ as the dielectric layer. Our main design strategy in achieving a maximum Q-factor with minimum spacing relies on the use of the distributed film capacitance as the LC tank circuit capacitance. The dominant parameter driving the resonance frequency shift is the on-chip capacitance change with mechanical deformation, allowing for strain measurement from the sensor without requiring additional circuitry. Although strain sensors using digital electronics [14,15] have been reported in the archival literature, the current work, to the best of our knowledge, represents the first account of an RF-based MEMS strain sensor in different architectures (circular geometries).

The following details using a circular architecture that better optimizes the aforementioned design aims. We compare two sensors with the same design parameters in rectangular and circular geometries shown in Figure 1. In both cases, the total size (W_c_ × L_c_) is 340 μm × 340 μm. In addition, both architectures have 2 turns (N), 60 μm wide metal width (w) and 10 μm wide spacing between coil segments (s). Their metal film thickness (t_metal_) is 0.1 μm while their dielectric film thickness (t_film_) is also 0.1 μm. The circular architecture has an effectively reduced total area compared to the rectangular geometry with the same dimensions. Thus, for the circular architecture, we obtain smaller film capacitance and coil inductance, yielding a higher f_o_. Also, we have lower coil resistance, lower loss, higher substrate resistance, and lower substrate capacitance. This produces a higher substrate loss factor and self-resonance factor, which is discussed in detail in [2]. As a result, with smaller spacing and higher f_o_ in the circular geometry, we achieve a higher inductor Q_ind_-factor (and thus a higher resonator Q-factor).

**Figure 2.**
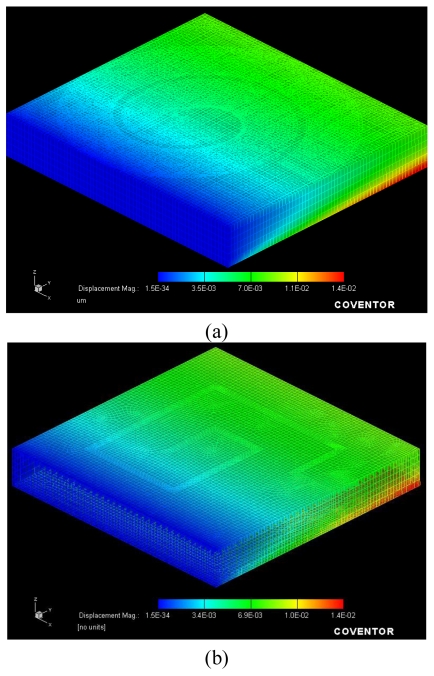
Coventorware simulations of the strain distribution of the deformed devices when a load of 1,960 N is applied from the bottom **(a)** in a circular geometry and **(b)** in a rectangular geometry. The z-direction is scaled down by a factor of 10 for a better view of the image.

We approach the increase in the resonance frequency shift from two perspectives. First, the deformation is equally effective in any direction, thanks to the isotropic geometry as depicted in Figure 2a. On the contrary, in a rectangular geometry, there is a preferential, anisotropic deformation, which dominates unilaterally (effective on only one side at a time) as illustrated in Figure 2b. In Figure 2, we can see that the maximum deformation of circular and rectangular shapes are the same but in rectangular geometry, one side is not deformed while in circular geometry, the whole geometry deformation is nearly the same. Therefore, the capacitance change in the circular case is higher than that in the rectangular case with the same starting initial capacitance value because the deformation acts to change the whole geometry. Hence, the associated resonance frequency shift is expected to be larger. Next, even if we have the same frequency shift ratio, Δf_o_/f_o_ (relative shift), the frequency shift is higher in the circular geometry since it possesses a higher f_o_. If we combine these two aspects, we have much higher shift for the circular case. Therefore, using the circular architecture, we expect to obtain a higher Δf_o_ and a higher sensitivity, e.g., defined as ∂f_O_/∂F (or as ∂f_O_/∂ε) with respect to the applied load (F) [or the induced strain (ε)]. Simulating S_21_ parameters for the rectangular and circular devices and their triplet configurations, we also obtain higher resonance frequencies and higher Q-factors for the circular geometry. Thus, we predict better performance with the circular architecture.

For the implementation of our devices, our fabrication process begins with deposition and patterning of a 0.1 μm thick metal contact layer (Au) on the substrate (Si), and subsequent deposition of a 0.1 μm thick dielectric layer (Si_x_N_y_), a cross-sectional view of which is shown in Figure 1c. We obtained the specific patterning with lithography and wet etching by hydrofluoric acid (HF). Subsequently, we metalized the open parts with 0.1 μm thick Au layer. Finally, another 0.1 μm thick final metal layer (Au) is deposited on top. The fabricated devices can be seen in Figures 1a,b,d,e.

## 3. Experimental Characterization and Analysis

To characterize our fabricated devices, we apply a point load to our devices in a controllable manner using the same method as in [3], where its schematic illustration is given in Figure 1f, and measure the device S_21_ parameter in response to the applied load. Thus, the change in resonance frequency and the Q-factor due to the applied load are determined. Our experimental set up includes an adjustable ultrafine-screw that can be adjusted to push towards the backside of the sensor. When the tip of this screw just touches the sensor backside, no load is applied, as verified by our reference strain gauge (made by Kyowa, Japan, with a gauge factor of 178). The screw is further twisted to apply load and induce strain. We confirmed the levels of strain induced with the position of the ultrafine-screw using our reference strain gauge.

For bioimplant sensing applications, there is an absolute requirement to measure and report strain remotely in the absence of wiring. Thus, for the current and future evaluations of this technology we need to measure and compare the telemetric performance of these sensors. To this end, we configure three resonators side by side on the same chip (in triplet configuration) and obtain an on-chip telemetry system. Although this on-chip system does not fully comply with the actual clinical application, it provides a robust methodology to compare different devices with respect to their telemetric operation. In this triplet configuration, the middle device serves as the sensor, with the lateral devices serving as the transmitter and receiver antennas. For calibration purposes, we measure the S_21_ parameter of the case where there are only transmitter and receiver antennas, and then measure the S_21_ parameters of our triplet configuration to obtain the resonance frequency and Q-factor. When the load is applied to the chip, the calibrations are again repeated with the same procedure as explained above to observe the changes in the resonance frequency and Q-factor. Also, using identical antennas guarantee to see the resonance frequency of the sensor since the working band of the antenna will definitely catch the resonance frequency of the sensor. Since the triplet method is used for easy coupling, using the antennas identical to the sensor makes our measurements further easier. A more detailed description of the triplet configuration operating principles is given in [3].

The human body presents a more complex environment compared to the lab environment. This side-by-side testing scenario (in triplet configuration) is an idealized one, as this configuration provides merely an *in vitro* characterization platform. Having characterized the operation of these sensors in a side-by-side configuration, our future research work will include performing animal model experiments. We anticipate that there will be differences in the performance of our sensor when placed in the *in vivo* environment. Specifically, we expect reduced sensitivity levels due to the complex nature of the *in vivo* measurement medium. We also expect that the circular architecture will greatly enhance some of the proposed application areas for this sensor due to the significantly improved sensing performance of the circular designs.

**Figure 3.**
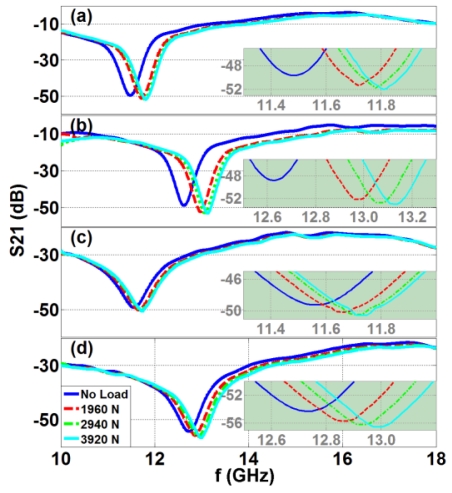
Experimental measurements of S_21_ parameters (dB) as a function of operating frequency (GHz) for **(a)** the single rectangular device, **(b)** the single circular device, **(c)** the rectangular triplet configuration, and **(d)** the circular triplet configuration under the applied loads of 1,960 N, 2,940 N, and 3,920 N, along with their zoom-in S_21_ parameters (dB) vs. operating frequency (GHz) (where the numbers of inset axes are grey colored) given around their resonances in the insets.

Figures 3a through 3d present S_21_ (in dB) as a function of operating frequency for the single rectangular, single circular, triplet rectangular, and triplet circular configurations, respectively. All of these figures also include a zoom-in view (in the inset) of the data around the resonance frequencies.

Table 1 lists the measured resonance frequencies in response to the applied loading, clearly showing that the resonance frequency increases with the applied force due to decreasing area, and hence, decreasing capacitance. Also, all of these experimental S_21_ data measured under zero external load are in agreement with our numerical simulations (in CST Microwave Studio).

In Table 1, we also present the resonance frequency changes. The resulting resonance frequency increase is higher for all of the circular device geometries as explained above. Since the area decrease is not linear and the capacitance is not linearly proportional to the resonance frequency, the resulting frequency increase is expectedly nonlinear. In addition, since the frequency shift is much higher in the circular cases compared to the rectangular cases, we observe higher relative shift and higher sensitivity for the circular cases in Table 1.

Table 1 also provides Q-factor data, which are observed to be high despite the relatively small chip sizes. These Q-factors are particularly higher in the circular case with a smaller area. The Q-factor is increased as the load magnitude is increased due to a lower C_film_, as discussed in [2] and [3]. The Q-factor also increases for the telemetric case of the circular case compared to the rectangular case. However, in the telemetric operation, due to coupling between resonators, the signal is decreased and Q-factors are reduced for both of the rectangular and circular cases compared to the single device cases. Our experimental apparatus can reproducibly apply a minimum strain of 81.5 microstrain, while the maximum strain is 172.8 microstrain. Therefore, it is not possible to make a direct measurement of the minimum detectable strain level for our sensors. Since the resolution of the network analyzer that we use in our experiments is 1 Hz (given the typical noise level in our experiments), we find the resolution of our sensors in the strain range across which they are tested by dividing this minimum detectable frequency to their sensitivities. From this calculation, we obtain 526.3 femtostrain for single rectangular device and 344.8 femtostrain for single circular device. These resolutions are better than those reported in [15].

Another interesting point for discussion is the hysteresis behavior. When different levels of external load are successively applied without allowing the mechanical setup to fully relax into the new loading conditions (typically in a time scale of minutes), we observe a memory effect and see a hysteresis in the experimental characterization of these sensors. The sensors in circular geometry exhibit a wider hysteresis loop as expected because they are more sensitive sensors, compared to those in the rectangular geometry. However, if one waits long enough (minutes) between successive force levels, no hysteresis is observed. The experimental data presented here is for the case of no hysteresis.

## 4. Conclusions

In summary, we have designed, fabricated, and experimentally characterized isotropic circular strain sensor resonators that allow for higher Q-factors with smaller spacing compared to rectangular designs. The circular architecture enables a significantly higher resonance frequency shift and sensitivity (both with respect to applied force and induced strain) because of its isotropic geometry. This results in a substantial improvement in the performance of these resonators for use as bioimplant strain sensors. With their promising properties and biocompatibility, our sensors are currently being investigated for the assessment of osseous fractures through monitoring the shift in the resonance frequency in response to the acting load.

## Figures and Tables

**Table 1.** Resonance frequencies, resonance frequency shifts, relative shifts, Q-factors, and sensitivities of our devices given as a function of changing load and induced strain levels.

**Load (N)**		**No load**	**1960**	**2940**	**3920**

**Microstrain**		**0**	**81.5**	**127.7**	**172.8**
**Single rect.**	f_o_ + Δf_o_ (GHz)	11.48	11.72	11.78	11.81
	Δf_o_ (MHz)	--	240	300	330
	Δf_o_/f_o_ (%)	--	2.1	2.6	2.9
	Q	59.979	70.348	74.324	76.000
	Sensitivity	0.0842 MHz/N or 1.9 MHz/microstrain			

**Single circ.**	f_o_ + Δf_o_ (GHz)	12.63	12.98	13.07	13.13
	Δf_o_ (MHz)	--	350	440	500
	Δf_o_/f_o_ (%)	--	2.8	3.5	4.0
	Q	72.461	91.667	93.025	93.786
	Sensitivity	0.1276 MHz/N or 2.9 MHz/microstrain			

**Triplet rect.**	f_o_ + Δf_o_ (GHz)	11.56	11.66	11.71	11.73
	Δf_o_ (MHz)	--	100	150	170
	Δf_o_/f_o_ (%)	--	0.9	1.3	1.5
	Q	33.801	36.347	38.243	39.231
	Sensitivity	0.0434 MHz/N or 1.0 MHz/microstrain			

**Triplet circ.**	f_o_ + Δf_o_ (GHz)	12.73	12.86	12.93	12.99
	Δf_o_ (MHz)	--	130	200	260
	Δf_o_/f_o_ (%)	--	1.0	1.6	2.0
	Q	44.033	50.431	53.364	55.442
	Sensitivity	0.063 MHz/N or 1.5 MHz/microstrain			
